# Long-term treatment with imatinib results in profound mast cell deficiency in Ph+ chronic myeloid leukemia

**DOI:** 10.18632/oncotarget.3074

**Published:** 2014-12-26

**Authors:** Sabine Cerny-Reiterer, Anja Rabenhorst, Gabriele Stefanzl, Susanne Herndlhofer, Gregor Hoermann, Leonhard Müllauer, Sigrid Baumgartner, Christine Beham-Schmid, Wolfgang R Sperr, Christine Mannhalter, Heinz Sill, Werner Linkesch, Michel Arock, Karin Hartmann, Peter Valent

**Affiliations:** ^1^ Ludwig Boltzmann Cluster Oncology, Medical University of Vienna, Austria; ^2^ Department of Internal Medicine I, Division of Hematology and Hemostaseology, Medical University of Vienna, Austria; ^3^ Department of Dermatology, University of Cologne, Cologne, Germany; ^4^ Department of Laboratory Medicine, Medical University of Vienna, Austria; ^5^ Department of Pathology, Medical University of Vienna, Austria; ^6^ Department of Pediatrics and Adolescent Medicine, Medical University of Vienna, Austria; ^7^ Institute of Pathology, Medical University of Graz, Austria; ^8^ Department of Internal Medicine, Division of Hematology, Medical University of Graz, Austria; ^9^ LBPA CNRS UMR8113, Ecole Normale Supérieure de Cachan, Cachan, France

**Keywords:** Mast Cells, KIT, Imatinib, Mast Cell Deficiency

## Abstract

Although mast cells (MC) play an important role in allergic reactions, their physiologic role remains unknown. In mice, several models of MC-deficiency have been developed. However, no comparable human model is available. We examined the in vitro- and in vivo effects of the KIT-targeting drug imatinib on growth and development of human MC. Imatinib was found to inhibit stem cell factor (SCF)-induced differentiation of MC in long-term suspension cultures (IC50: 0.01 μM). Correspondingly, long-term treatment of chronic myeloid leukemia (CML) patients with imatinib (400 mg/day) resulted in a marked decrease in MC. In patients with continuous complete molecular response during therapy, bone marrow MC decreased to less than 5% of pre-treatment values, and also serum tryptase concentrations decreased significantly (pre-treatment: 32.0±11.1 ng/ml; post-therapy: 3.4±1.8, p<0.01). Other myeloid lineages, known to develop independently of KIT, were not affected by imatinib-therapy. Imatinib also produced a substantial decrease in MCdevelopment in mice. However, no clinical syndrome attributable to drug-induced MC-deficiency was recorded in our CML patients. Together, imatinib suppresses MC production in vitro and in vivo. However, drug-induced MC depletion is not accompanied by adverse clinical events, suggesting that MC are less relevant to homeostasis in healthy tissues than we assumed so far.

## INTRODUCTION

Mast cells (MC) are bone marrow (BM) stem cell-derived, tissue-fixed, multipotent effector cells of the immune system [1-8]. These cells store a number of vasoactive and immunomodulating substances in their granules and express high-affinity receptors for IgE [1-10]. During an allergic reaction, MC release their immunomodulating and vasoactive mediators and thereby contribute to the clinical symptoms of allergy [1-5,9,10]. In addition, MC have been considered to play an important role in other inflammatory reactions, in natural host defence, and in tissue homeostasis [1-6].

MC are a rich source of histamine, various proteases including tryptase and chymase, proteoglycans, and various cytokines such as tumor necrosis factor-alpha [1-5,11-15]. Moreover, MC exhibit a unique profile of profibrinolytic and anti-thrombotic substances, such as tissue-type plasminogen activator (tPA), urokinase receptor, and heparin [16-18]. It has also been described that MC increase in number and accumulate around thrombosed vessels, and that MC supernatants can dissolve thrombotic clots [16-20]. Finally, it has been described, that india ink-induced fatal thrombosis occurs more frequently in MC-deficient mice than in control animals [21]. Other studies have shown that MC-deficient mice are more susceptible to fatal bacterial infections compared to control animals [22,23]. All these observations suggest that MC may fulfil important functions in i) the immune system and ii) in the vascular repair system under pathologic conditions. In addition, MC have been considered to play a role in normal tissue homeostasis. However, the exact role of MC in normal healthy (physiologic) tissues has not been described yet. Whereas several models of MC deficiency are available in the murine system [1,21-25], no comparable model of human MC deficiency is available.

A number of previous studies have shown that the ligand of the KIT tyrosine kinase receptor, stem cell factor (SCF), promotes the *in vitro* growth and development of MC [26-29]. In line with this concept, MC and MC progenitors express KIT throughout their development [28-30]. Moreover, it is well accepted that SCF-deficient mice and KIT-deficient mice exhibit profound MC deficiency [24,25]. SCF-dependent development of MC from their immature progenitor cells is a long-lasting process that takes several months [26-29]. In addition, mature MC in various organs are considered to be long-lived cells that can persist in local tissue sites for several years [31].

During the past decade, the tyrosine kinase inhibitor (TKI) imatinib has been successfully used for the treatment of patients with BCR/ABL1+ chronic myeloid leukemia (CML) and for the treatment of FIP1L1/PDGFRA+ chronic eosinophilic leukemia (CEL) [32-36]. In fact, imatinib is a potent inhibitor of BCR/ABL1 and FIP1L1/PDGFRA. In many patients with CML, long-term disease-free survival and major (MMR) or even complete molecular responses (CMR) are obtained [32-35]. In addition, imatinib is a highly potent inhibitor of the KIT tyrosine kinase [37]. However, the effect of imatinib on KIT-dependent cells, especially tissue MC, remains at present unknown. In the current study, we asked whether long-term treatment of CML patients with imatinib is associated with MC deficiency.

## RESULTS

### Imatinib inhibits SCF-induced differentiation of human MC in long-term culture

**Figure 1:**
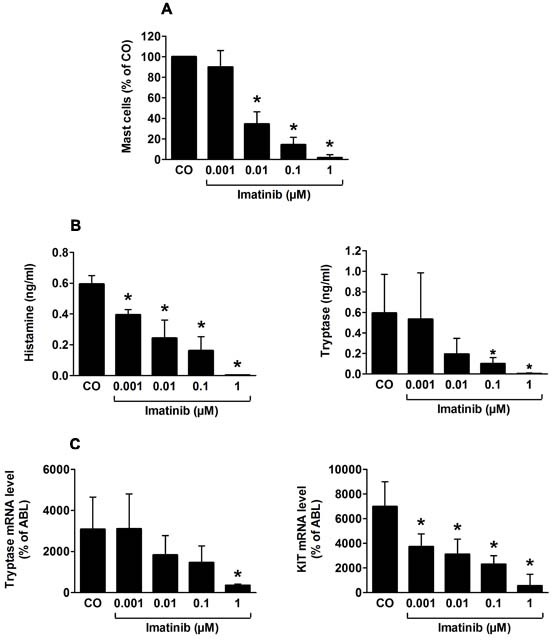
Imatinib inhibits SCF-induced *in vitro* differentiation of human mast cells A: Isolated cord blood MNC (1×10^6^/ml) were cultured in 24-well plates in RPMI medium containing 10% FCS, IL-6 and SCF (each 100 ng/ml) in the presence or absence (CO) of various concentrations of imatinib (0.001-1 μM) at 37°C for 28 days. Thereafter, the total numbers of mast cells (MC) per well were determined by measuring total cell counts and the percentage of MC on Giemsa-stained slides. Cells were counted using an Olympus AX-1 microscope equipped with a 100x/1.35 UPlan-Apo objective lens. Results represent the mean±S.D. from 3 independent experiments. Asterisk: p<0.05. B: Total histamine levels per well assessed by RIA (left panel) and total tryptase levels assessed by FEIA (right panel) were determined on day 28. Results represent the mean±S.D. from 3 independent experiments. Asterisk: p<0.05. C: Expression of tryptase mRNA levels (left panel) and KIT (CD117) mRNA levels (right panel) determined by qPCR on day 28. Results show tryptase and KIT mRNA expression levels as percent of ABL mRNA levels, and represent the mean±S.D. from 3 independent experiments. Asterisk: p<0.05.

It is generally appreciated that SCF promotes the development and differentiation of human MC [1-3,26-28]. In the present study, we were able to confirm the MC growth-stimulating effect of SCF on CB-derived MC precursor cells in long-term suspension cultures. In particular, as assessed by Wright-Giemsa staining, substantial numbers of MC were detectable in SCF-supplemented cultures on day 28, whereas in cultures maintained in control medium (without SCF), no MC were detected (not shown). In addition, cellular histamine- and tryptase levels were measured in CB precursor cells cultured in the presence of SCF. In these cultures, imatinib was found to inhibit SCF-dependent differentiation of MC in a dose-dependent manner (Figure 1). The growth-inhibitory effects of imatinib on MC development were demonstrable by morphological examination (Figure 1A) as well as by measuring total histamine and total tryptase levels in cultured cells (Figure 1B). In addition, imatinib was found to inhibit SCF-induced expression of tryptase mRNA and KIT mRNA in long-term suspension cultures (Figure 1C). Together, these data show that imatinib exerts profound inhibitory effects on SCF-dependent development and differentiation of human MC *in vitro*.

### Long-term treatment of CML patients with imatinib is associated with a substantial decrease in the numbers of BM MC

**Figure 2:**
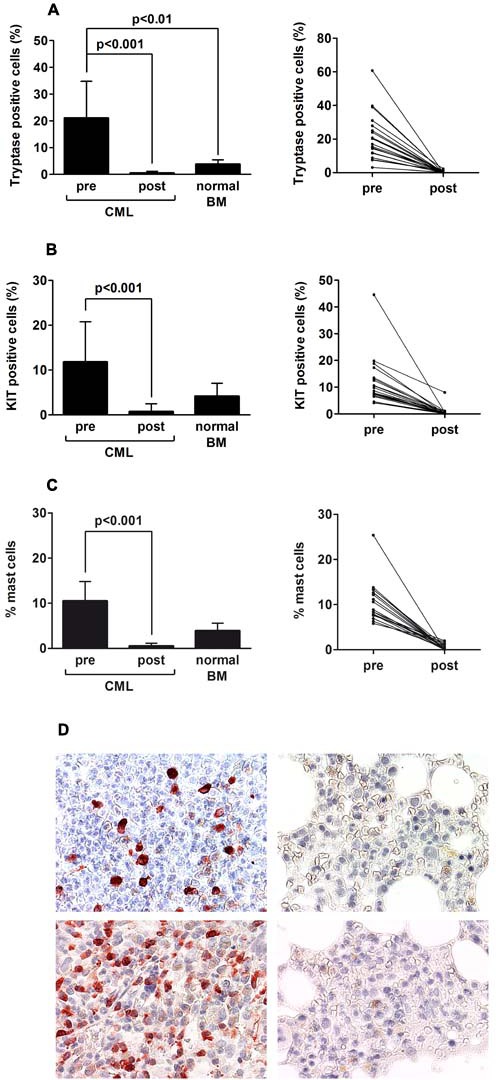
Imatinib induces mast cell deficiency in the bone marrow of patients with CML Bone marrow (BM) biopsy material was obtained from patients with CML (n=23) at diagnosis and at the time of major or complete molecular response and at least 2 years on therapy with imatinib (400 mg/day). In addition, control BM section from 5 patients were examined. Serial sections were prepared from paraffin-embedded BM specimens and stained with antibodies against tryptase (A) and against KIT (B) by indirect immunohistochemistry as well as by Giemsa-staining (C). The percentages of tryptase+ mast cells (MC) and KIT+ MC relative to all nucleated BM cells (500 cells counted), was determined using an Olympus AX-1 microscope equipped with 100x/1.35 UPlan-Apo objective lens. Results in the left panels represent the mean±S.D. (percent-values) from all donors before and after therapy, and a comparison to normal control BM samples (n=5). The right panels show the percentages of tryptase+ MC and KIT+ MC in each individual patient before and after therapy. In C, the numbers of MC was determined by Giemsa-staining (percent of all nucleated BM cells) using an Olympus AX-1 microscope. Results in the left panel represent the mean±S.D. from all donors before and after therapy, and a comparison to normal control BM samples (n=5). The right panel shows the percentages of MC in each individual patient. D: Examples of BM sections stained for tryptase (upper panels) and KIT (lower panels) at diagnosis (upper and lower left panels) and at the time of re-investigation (upper and lower right panels) by indirect immunohistochemistry. Cells were analyzed using an Olympus AX-1 microscope equipped with 40x/0.85 UPlan-Apo objective lens. Images were taken using an Olympus DP21 camera and adjusted by Adobe Photoshop CS2 software Version 9.0 (Adobe Systems).

The numbers of tryptase+ cells and the numbers of KIT+ cells in the BM of newly diagnosed patients with CML exceeded the numbers of tryptase+ and KIT+ cells detected in the normal BM (Figure 2A and 2B) suggesting an expansion of clonal tryptase+ cells. After efficacious long-term treatment with imatinib, defined by MMR (or CMR) for at least 24 months, the numbers of tryptase+ cells and the numbers of KIT+ cells decreased significantly (p<0.001) compared to pre-treatment values (Figure 2A and 2B). In addition, we found that the numbers of tryptase+ cells and KIT+ cells in the BM of our long-term-treated patients were even lower when compared to the numbers of tryptase+ cells or KIT+ cells (MC) detectable in the normal BM (p<0.01) (Figure 2A and 2B). The MC-depleting effect of imatinib was confirmed by Giemsa-staining (Figure 2C). Figure 2D shows examples of immunohistochemical stains performed with antibodies against tryptase and KIT in BM sections obtained from patients with CML before and after therapy with imatinib. We also confirmed that long-term treatment with imatinib results in a complete depletion of clonal cells in the BM. In fact, in all MMR patients tested, BCR/ABL1 mRNA levels in BM mononuclear cells were <0.1% by qPCR, similar to peripheral blood BCR/ABL levels (not shown). Since MC development is a long-lasting process and MC may persist in normal tissues for several years [31] we also examined BM sections of patients treated with imatinib for less than 1 year. In this cohort of patients (n=6) the numbers of tryptase+ cells and KIT+ cells (MC) also decreased slightly compared to pre-treatment values, but the decrease was not significant, and the concentrations of tryptase+ cells and KIT+ cells in the BM were comparable to that found in the normal BM (Supplemental Figure S1).

**Table 1:** Patients´ characteristics at diagnosis

Pat.No.	Gender	Age (y)	Phase	WBC (G/L)	Hb (G/dL)	Plt (G/L)	PB Basophils (%)	PB Blasts (%)	PB Eosinophils (%)	BM Basophils (%)	BM Blasts(%)	Cytogenetic	BCR/ABL1 PB (%)
1	m	29	CP	375.3	10.6	193	2	2	1	2	2	46,XY,t(9;22)(q34;q11)	63.08
2	f	58	CP	70.8	10.8	1722	11	3	2	8	7	46,XX,t(9;22)(q34;q11)	53.00
3	f	36	CP	193.8	11.5	222	4	1	2	<1	2	46,XX,t(9;10;22)(q34;q22;q11)	100.00
4	m	37	CP	381.0	9.7	275	5	3	4	4	3	46,XY,t(9;22)(q34;q11)	18.44
5	m	60	CP	171.0	12.1	305	2	1	0	2	<1	46,XY,t(9;22)(q34;q11)	pos. qualitat.
6	m	64	CP	342.0	8.9	478	7	1	2	6	3	46,XY,t(9;22)(q34;q11)	14.30
7	f	63	CP	43.5	11.7	265	19	1	3	10	4	46,XX,t(9;22)(q34;q11)	7.50
8	m	47	CP	55.7	13.9	250	1	1	0	1	3	46,XY,t(9;22)(q34;q11)	34.40
9	f	42	CP	61.2	13.5	319	3	0	1	<1	<1	46,XX,t(9;22)(q34;q11)	61.60
10	m	60	CP	29.9	15.7	252	6	0	3	2	1	46,XY,t(9;22)(q34;q11)	62.70
11	m	37	BP	104.3	11.4	137	6	17	10	10	19	46,XY,t(9;22)(q34;q11)	76.00
12	m	55	CP	46.7	11.9	336	9	1	1	<1	<1	46,XY,t(9;22)(q34;q11)	63.00
13	f	57	CP	175.2	9.8	216	4	4	7	3	2	46,XX,t(9;22)(q34;q11)	82.60
14	f	63	CP	49.1	13.0	385	2	0	1	0	1	46,XX,t(8;9;22)(q13;q34;q11)	pos. qualitat
15	f	59	CP	69.1	12.7	297	2	0	4	0	1	46,XX,t(9;22)(q34;q11)	pos. qualitat
16	m	40	CP	86.2	13.9	134	4	0	1	n.d.	n.d.	46,XY,t(9;22)(q34;q11)	19.20
17	m	58	CP	170.0	9.6	506	1	2	4	2	0	46,XY,t(9;22)(q34;q11)	49.00
18	m	55	CP	142.0	11.9	465	7	7	6	8	5	46,XY,t(9;17;22)(q34;q25;q11)	15.80
19	m	58	CP	183.0	8.5	397	15	10	6	7	4	46,XY,t(9;22)(q34;q11)	pos. qualitat
20	m	42	CP	147.5	10.2	528	3	0	3	<1	1	46,XY,t(9;22)(q34;q11),del(11)(q23)	88.10
21	f	36	CP	96.1	14.3	800	11	1	4	2	2	46,XX,t(9;22)(q34;q11)	30.00
22	f	44	CP	103.4	11.8	569	3	1	2	1	1	46,XX,t(9;22)(q34;q11)	13.00
23	m	50	CP	33.6	11.4	351	1	0	0	<1	2-3	46,XY,t(9;22)(q34;q11)	36.50
24	m	43	BP	67.1	13.7	1460	1	2	3	0	18	n.d.	13.10
25	f	57	CP	124	13.6	549	0	1	0	0	0	n.d.	13.40
26	f	65	BP	1.65	10.5	48	1	0	5.5	1	35	n.d.	19.00
27	f	61	CP	15.5	13	870	5	0	3	0	0	n.d.	15.30
28	m	59	CP	98.6	13.3	338	4	1	2	0	0	n.d.	37.20
29	m	68	CP	20.2	15.2	417	10	0	1	0	2	n.d.	20.10

Abbreviations: y, year; WBC, white blood count; Hb, hemoglobin; Plt, platelets; BM, bone marrow; PB, peripheral blood; m, male; f, female; CP, chronic phase; BP, blast phase; pos. qualitat., BCR/ABL1+ by qualitative PCR; n.d., not determined.

### Tryptase mRNA- and KIT mRNA levels during treatment with imatinib

**Figure 3:**
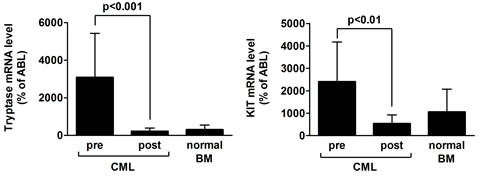
Tryptase mRNA and KIT mRNA levels before and after treatment with imatinib Isolated bone marrow (BM) cells obtained from 12 CML patients before therapy and after at least 2 years of therapy with imatinib (at the time of major or complete molecular response) were subjected to RNA isolation and qPCR using primers specific for tryptase, KIT, and ABL/EXON1. Results show tryptase mRNA expression levels (left panel) and KIT mRNA expression levels (right panel) as percent of ABL/EXON1 mRNA levels. Results represent the mean±S.D. from all donors in each group. Asterisk: *p<0.05.

In order to confirm imatinib-induced MC deficiency in our CML patients, we examined MNC derived from aspirated BM samples by qPCR using primers specific for mast cell tryptase and KIT. As visible in Figure 3, tryptase mRNA levels and KIT mRNA levels decreased significantly during successful long-term treatment with imatinib in all patients examined. In these patients, tryptase mRNA levels and KIT mRNA levels were found to be even lower when compared to that found in normal BM cells (p<0.01) (Figure 3).

### Successful long-term treatment with imatinib induces a systemic decrease in MC

**Figure 4:**
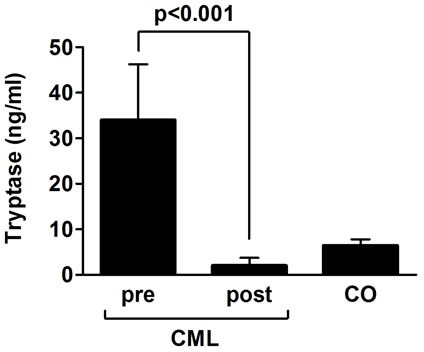
Successful treatment with imatinib induces systemic mast cell deficiency Total serum tryptase levels were measured in 10 patients with CML at diagnoses and in the same patients after at least 2 years of treatment with imatinib at the time of major or complete molecular response. In addition, serum tryptase levels were measured in 10 healthy controls. Tryptase levels were determined by fluoroenzyme-immunoassay. Results represent the mean±S.D. of all donors in each group.

In a next step, we asked whether the effect of imatinib on MC is a systemic effect. In order to address this question, we measured serum tryptase levels before and after treatment with imatinib in a subset of our patients, and compared post-treatment levels to pre-treatment levels and to tryptase levels in healthy controls. As visible in Figure 4, tryptase levels at diagnosis were found to be higher compared to tryptase levels in healthy controls (p<0.01). After treatment with imatinib, serum tryptase levels decreased significantly in all patients when compared to pre-treatment levels (p<0.001) or to tryptase levels in controls (p<0.05) (Figure 4). In several cases, tryptase decreased to very low or even undetectable levels (not shown). To exclude that the low serum tryptase level resulted from deactivation of MC rather than depletion of MC, we also performed activation experiments. In these experiments, we found that imatinib neither blocks IgE-dependent nor SCF-mediated release of histamine in CB precursor-derived MC or human lung MC (not shown). Together, these data provide evidence that long-term treatment with imatinib results in a substantial systemic decrease in MC numbers. Finally, we examined the influence of long-term imatinib therapy on growth and differentiation of other blood cells. However, no significant changes in white blood cell numbers were found when comparing long-term treated CML patients with normal blood counts (Table 3). Finally, we were unable to record any adverse events possibly related to MC-depletion in our CML patients treated with imatinib, even when MC numbers had decreased to very low levels. In particular, no increased frequency in thromboembolic events, severe bacterial or fungal infections or bleedings, were recorded (Table 4). We were also unable to detect an increased rate of secondary cancer or leukemias in our imatinib-treated patients. The most frequent adverse event was mild to moderate (facial/lid) edema, confirming previous observations [32-34].

### Treatment with imatinib induces a marked decrease in MC numbers in mice

**Figure 5:**
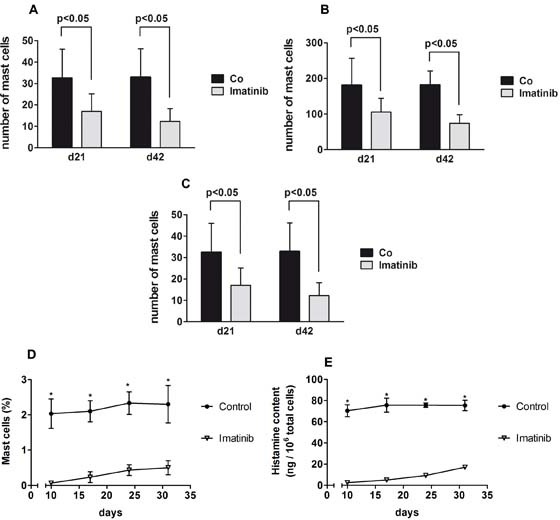
Effects of imatinib on mast cell (MC) numbers in C57BL/6J and BALB/c mice A-C: C57BL/6J mice were treated with imatinib (60 mg/kg per day) or vehicle control (CO) by intraperitoneal (i.p.) injection for 42 days (5 mice per group). Animals were sacrificed on days 21 or 42 and the percentages of MC in the back skin (A), ear skin (B) and gastric mucosa (C) were determined by staining with toluidine blue (TB) as described in the text. In A, B and C, the numbers of MC was determined by TB staining using a Leica DM 4000 B microscope (Solms, Germany) equipped with a HC Plan Apo 20x/0.70 objective lens. Results are expressed as numbers of TB+ cells per HPF using Discus software (Discus, Columbus, Ohio, USA) and represent the mean±S.D. of all mice per group. D-E: Imatinib-induced *in vivo* depletion of MC in the peritoneum of BALB/c mice. D: Mice were treated with imatinib (25 mg/kg twice daily by i.p. injection) or vehicle alone for 10 days. Peritoneal fluid was recovered in each group of mice (n=3) at various time points (days 10, 17, 24 and 31). The percentage of MC among total peritoneal cells was determined in each animal by TB staining using an Olympus BX51 microscope equipped with a UPlanFI 20x/0.50 objective lens. Results are expressed as mean±S.D. of all mice in each group (imatinib- or vehicle-treated mice). Asterisk: p<0.001. E: Peritoneal fluid samples were adjusted at 10^6^ per mL and subjected to freeze-thawing. Then, cellular histamine levels were determined. Results are expressed as mean concentrations of histamine (ng/10^6^ total peritoneal cells) and represent the mean±S.D. of all mice per group (imatinib- or vehicle-treated animals). Asterisk: p<0.001.

To confirm the effect of imatinib on MC numbers *in vivo* in mice, two different mouse strains were employed. Intraperitoneal injection of imatinib (60 mg/kg/day) in C57BL/6J mice for 42 days resulted in a time-dependent decrease in MC in various organ-sites, including the back-skin, ear-skin, and the gastrointestinal tract, i.e. gastric mucosa (Figure 5A-C). MC numbers in the back-skin decreased from 36.7±10.1 per high power field (control mice on day 42) to 15.7±4.6 per high power field in imatinib-treated animals on day 42 (p<0.05), and a similar decrease was observed in the ear-skin and in the intestinal mucosa of imatinib-treated C57BL/6J mice (Figure 5). The MC-depleting effect of imatinib was also examined in BALB/c mice. In these experiments, mice were treated with 25 mg/kg imatinib (i.p. injection) twice daily for 10 days. Treatment of mice with imatinib resulted in a substantial decrease in the percentage of MC and a significant decrease in cellular histamine levels in the peritoneum compared to vehicle control (Figure 5D and 5E). Although BALB/c mice were only treated for 10 days, the effect of imatinib was demonstrable until day 31 in all animals (Figure 5D and 5E). Histamine levels on day 31 amounted to 70.33±5.69 ng/10^6^ cells in vehicle-control-treated animals, compared to only 2.43±0.76 ng/10^6^ cells in imatinib-treated mice (p<0.01).

**Table 2:** Patient´s characteristics at the time of re-investigation (patients in MMR or CMR after 2 to 10 years)

Pat.No.	Gender	Age (y)	Time(y) after Diagnosis	WBC (G/L)	Hb (G/dL)	Plt (G/L)	PB Basophils (%)	PB Blasts (%)	BM Basophils (%)	BM Blasts (%)	Cytogenetic	BCR/ABL1 PB (%)
1	m	31	2	2.93	14.3	140	0	0	0	0	46,XY	0.061
2	f	70	11	4.24	11.2	68	0	0	1	1	46,XX	0.020
3	f	40	3	2.79	12.6	122	0	0	<0,5	1	n.d.	0.009
4	m	46	8	6.54	14.2	204	1	0	<1	2	46,XY	0.092
5	m	67	7	5.85	12.6	157	1	0	<1	1	46,XY	0.000
6	m	71	7	4.13	12.0	134	0	0	<1	1	46,XY	0.031
7	f	71	8	4.76	12.2	232	0	0	<1	1	46,XX	0.000
8	m	50	7	4.94	13.7	176	1	0	<1	1	46,XY	0.018
9	f	47	5	4.89	11.3	194	1	0	<1	1	46,XX	0.013
10	m	63	3	5.37	14.8	179	0	0	<1	1	46,XY	0.002
11	m	43	5	4.65	11.7	147	1	0	<1	1	46,XY	0.013
12	m	60	5	4.77	13.0	254	1	0	<1	1	46,XY	0.000
13	f	63	6	4.63	12.6	206	0	0	<1	1	46,XX	0.025
14	f	74	10	5.84	11.4	256	1	0	<1	1	46,XX	0.055
15	f	69	10	4.23	10.7	225	0	0	<1	1	46,XX	0.005
16	m	48	7	7.29	15.6	177	2	0	<1	1	46,XY	0.007
17	m	71	12	4.58	15.1	108	0	0	<1	2	n.d.	0.000
18	m	64	9	5.38	12.6	139	0	0	<1	1	46,XY	0.011
19	m	69	11	6.22	12.8	120	0	0	<1	<1	46,XY	0.013
20	m	48	6	5.73	12.5	302	0	0	<1	1	46,XY	0.016
21	f	47	11	5.62	12.5	230	0	0	<1	1	46,XX	0.000
22	f	48	4	2.94	11.8	238	2	0	1	<1	46,XX	0.000
23	m	52	2	5.39	14.1	173	0	0	<1	2	46,XY	0.000
24	m	44	1	3.01	9.4	34	1	0	0	0	n.d.	0.000
25	f	58	1	7.1	13.4	261	0	0	0	0	n.d.	0.403
26	f	65	0.5	4.19	13.1	50	0	0	2.1	0	n.d.	0.023
27	f	62	1	8.34	12.4	353	1	0	3	0	n.d.	0.140
28	m	59	0.4	3.6	13	77	0	0	1	0	n.d.	0.860
29	m	69	1	6.6	14.3	325	0	0	5	0	n.d.	0.577

Abbreviations: y, year; WBC, white blood count; Hb, hemoglobin; Plt, platelets; BM, bone marrow; PB, peripheral blood; m, male; f, female, n.d., not determined

**Table 3:** Differential counts at the time of at least major molecular response (MMR)

	absolute numbers (G/L)	reference values (G/L)	relative numbers (%)	reference values (%)
Neutrophils	2.87±0.84	2.0-7.5	57.61±9.40	50-75
Lymphocytes	1.51±0.50	1.0-4.0	31.13±8.85	25-40
Monocytes	0.36±0.15	0.0-1.2	7.43±2.74	0-12
Eosinophils	0.17±0.17	0.0-0.4	3.30±2.98	0-4
Basophils	0.03±0.04	0.0-0.1	0.47±0.67	0.0-1.0

Leukocyte counts were determine in all CML patients (n=23) before the start of therapy with imatinib and during therapy at the time of (at least) MMR. Differential counts were examined on Wright-Giemsa-stained blood smears.

**Table 4:** Adverse events possibly related to mast cell deficiency or mast cell activation

Pat No	Throboembolic Events	Bacterial or Fungal Infection	Major Bleeding	Exanthema/Allergy	Edema	Other Symptoms
1	-	-	-	Exanthema	-	-
2	-	-	-	-	lid	-
3	-	-	-	-	-	-
4	-	-	-	-	lid	-
5	-	-	-	-	-	-
6	-	-	-	-	-	diarrhea
7	-	-	-	exanthema	leg	diarrhea
8	-	-	-	-	-	-
9	-	-	-	exanthema	lid	diarrhea
10	-	-	-	-	-	-
11	-	-	-	-	-	-
12	-	-	-	-	leg	-
13	-	-	-	-	-	-
14	-	urinary infection	-	-	leg	-
15	-	-	-	-	-	-
16	-	-	-	exanthema	-	-
17	-	bronchitis	-	-	-	-
18	-	-	-	-	lid	-
19	-	-	-	-	lid,leg	diarrhea
20	-	-	-	-	-	diarrhea
21	-	-	-	-	-	-
22	-	-	-	-	lid	-
23	-	-	-	-	-	-

Clinical symptoms were examined in a retrospective manner, lid, eyelid.

## DISCUSSION

Mast cells are key effector cells of the immune system and have been implicated in the pathogenesis of allergic and other immunologic disorders [1-8]. However, whereas the role of MC in certain disease models is well established, it remains unclear whether MC also play a role as physiologic cells in healthy tissues. This is an important question in basic science as well as in clinical practice because several different drugs are known to block MC function, and some of the recently developed KIT-blocking TKI may even lead to a decrease in MC numbers. In this study, we show that successful long-term treatment of CML patients with imatinib results in a profound decrease in MC numbers. To the best of our knowledge this is the first description of drug-induced depletion of MC in the human system. In addition, our data show that imatinib inhibits SCF-induced and thus KIT-dependent development of MC from their CB-derived progenitor cells. However, despite inducing a marked decrease in MC numbers, long-term treatment of CML patients with imatinib was not accompanied by major adverse events or specific symptoms, suggesting that MC may play a less important biological role in steady state homeostasis of healthy tissues than has so far been assumed.

The ligand of KIT, SCF, is a well-known differentiation factor for human MC [1-3,7,26-29]. In the present study, we were able to confirm that SCF induces MC differentiation in CB-derived progenitor cells [38-40]. In these cultures, we applied a combination of SCF and IL-6 which may be equally potent or even a more potent stimulus of MC differentiation compared to SCF alone [26-29,38-40]. In this assay, imatinib was found to suppress cytokine-induced development of human MC in a dose-dependent manner, with IC_50_ values of about 10 nM which is a pharmacologically relevant drug concentration. Complete suppression of MC development was seen at 1 μM of imatinib. These data suggest that imatinib blocks cytokine-dependent differentiation of MC. Since *in vivo* development of MC is also considered to depend primarily on SCF activation of KIT in MC progenitors, this observation may have clinical implications and may explain why MC decrease in number during treatment with imatinib. However, MC development from their progenitors is a long-lasting process [26-29] and mature MC may persist in local tissue sites for several months to years. Based on observations made in transplanted patients, MC repopulation from their progenitors may take at least 1 year [31].

Therefore, we decided to examine MC numbers in the BM of our CML patients after 2 years following successful treatment with imatinib in this study. Since MC might also derive from CML stem- and progenitor cells in such patients, a second requirement was that imatinib had produced at least MMR, and that these patients were in MMR or CMR for at least one year. We found that successful long-term treatment with imatinib produces a marked decrease in the numbers of BM MC in these patients. Notably, the numbers of MC decreased from elevated pre-treatment levels to low or even undetectable levels in post-therapy BM samples. The decrease in MC in BM sections was demonstrable by staining for MC tryptase and KIT as well as by Giemsa staining. Moreover, we were able to show that imatinib therapy leads to a substantial decrease in tryptase mRNA and KIT mRNA levels in BM cells. It is noteworthy to state that MC concentrations in the BM not only decreased significantly during imatinib when compared to pre-treatment levels in our CML patients, but also when compared to MC numbers recorded in the BM of normal healthy controls. Collectively, these data suggest that long-term treatment with imatinib not only resulted in a markedly suppressed developent of normal MC but also in suppression of growth of clonal (BCR/ABL+) MC progenitors, which was confirmed by qPCR.

To demonstrate that the decrease in MC numbers in the BM is a long-lasting process and time-dependent, we also examined a smaller cohort of patients after one year of treatment with imatinib. In these patients, no significant decrease in MC numbers was found when compared to pre-treatment values. We also examined other cell types in the BM and in the peripheral blood. However, we were not able to detect a significant decrease of any other cell type, including basophils, eosinophils or monocytes in our imatinib-treated CML patients, regardless of the time point examined. This observation is consistent with the notion that MC development is specifically dependent on KIT and thus SCF, whereas the development of other hematopoietic cell types is dependent on other cytokines, but not SCF [1-7,26-29]. These data also support the concept that MC are not derived from blood monocytes or blood basophils as evidenced by our previous studies [41].

MC are well-known to reside in various tissues and organ systems [1-8]. Therefore, we were interested to learn whether imatinib induces local MC deficiency in the BM or a systemic or even global MC deficiency. Since we were unable to examine all organ systems by biopsy in our CML patients, we decided to measure serum total tryptase levels before and after (during) successful therapy with imatinib. In this regard it is noteworthy to state that the basal tryptase level usually results from a constant release of the enzyme from MC in various tissues [42,43]. Therefore, the basal tryptase level is a generally accepted parameter of the total body burden of MC in healthy controls. During anaphylaxis, serum tryptase levels may transiently increase, and in patients with systemic mastocytosis, serum tryptase levels are persistently elevated [43,44]. We were interested to learn whether imatinib would directly block the spontaneous or IgE-dependent release of tryptase from human MC. However, imatinib failed to block spontaneous or anti-IgE-induced release of histamine or tryptase in human MC.

In patients with CML, elevated serum tryptase levels were detected at diagnosis in a subset of patients, confirming our previous data [45,46]. However, in contrast to mastocytosis, elevated tryptase levels in CML apparently result from an increased production and release in immature basophils [45,46]. Successful long-term treatment of our CML patients with imatinib was found to result in a significant decrease in serum tryptase levels. These tryptase levels were found to be even lower compared to that found in healthy controls. Collectively, these data suggest that long-term therapy with imatinib produces not only a substantial decrease in clonal tryptase+ cells (MC and immature basophils) in the BM but also a markedly reduced systemic production of normal MC in various organ systems. An alternative explanation would be that imatinib inhibits the basal secretion of tryptase from MC. However, this possibility could be eliminated as outlined above. Another explanation would be that a selective depletion of MC in the BM is sufficient to decrease serum tryptase levels. However, this possibility seems unlikely. In fact, the numbers of MC in the lungs, skin, and in the gastrointestinal tract exceed MC numbers in the BM by far, so that a selective decrease in BM MC should not lead to a visible decrease in tryptase levels.

To provide definitive evidence for a systemic effect of imatinib on MC development, we employed two different mouse strains, namely C57BL/6J and BABL/c. In both models, animals were treated with imatinib by i.p. injection, and in both types of mice, the drug produced a substantial decrease in MC numbers in all organs tested, including the skin, peritoneum and the gastric mucosa. In contrast to the human system, imatinib-induced MC depletion in mice was already seen after a few weeks, which may be explained by the fact that the development of murine MC occurs within a (much) shorter time interval compared to SCF-dependent development of human MC.

MC are well known to fulfil important functions in the immune system and in allergic reactions [1-9]. Moreover, MC have been described to play a potential role as repair cells during infections and during or/and after a thromboembolic event [16-23]. Therefore, we were interested to learn whether imatinib-induced MC deficiency would predispose for certain adverse events, such as thromboembolic events, bacterial or fungal infections or cancer development. However, although investigated thoroughly, we were unable to detect an increased frequency of bacterial or fungal infections, cancer, or thromboembolic events in our imatinib-treated patients with CML.

In conclusion, our study shows that the KIT-blocking TKI imatinib produces a profound decrease in MC in mice as well as a decrease in MC in patients with Ph+ CML. However, imatinib-induced MC depletion is not accompanied by specific adverse events or symptoms, suggesting that MC are less important cells in the homeostasis of normal healthy tissues than has been assumed so far. Finally, imatinib-induced MC depletion is lineage-specific and not accompanied by a substantial decrease in other leukocytes, confirming that MC are derived from a separate stem cell pool but not from mature blood basophils or blood monocytes.

## PATIENTS AND METHODS

### Patients´ characteristics and bone marrow sampling

Bone marrow biopsy specimens were obtained from 29 patients with Ph+ CML. Diagnoses were established according to WHO criteria [47]. The patients´ characteristics are shown in Table 1. Informed consent was obtained in all patients before BM or blood was obtained. All studies were approved by the ethics committee of the Medical University of Vienna. In 23 patients, biopsy material was obtained at diagnosis and at the time of major (MMR) or complete (CMR) molecular response and at least two years of therapy with imatinib (400 mg/day). In a smaller cohort of patients (n=6), BM samples were examined at diagnosis and within the first year of treatment. All patients were examined for the development of adverse events during treatment with imatinib. Furthermore, we examined normal/reactive bone marrow (n=5) as control.

### Reagents

The monoclonal antibody (mAb) G3 (IgG1) against tryptase was purchased from Chemicon (Temecula, CA), a rabbit polyclonal antibody against KIT (CD117) from Dako (Glostrup, Denmark), biotinylated anti-rabbit IgG, anti-mouse IgG and Vectastain Universal ABC-AP Kit from Vector Laboratories (Burlingham, CA), and 3-amino-9-ethylcarbazole (AEC) from Sigma (St.Louis, MO, USA). Imatinib was kindly provided by Dr.E.Buchdunger and Dr.P.Manley (Novartis Pharma AG, Basel, Switzerland). Recombinant human (rh) stem cell factor (SCF) was from Strathmann Biotech (Hannover, Germany) and rh interleukin-6 (IL-6) from Novartis Pharma AG. RPMI 1640 medium and fetal calf serum (FCS) were purchased from PAA laboratories (Pasching, Austria) and a histamine radioimmuno-assay (RIA) from Immunotech (Marseilles, France). Serum and cellular tryptase levels were measured by fluoroenzyme-immunoassay (FEIA, Thermo Fisher Scientific, Uppsala, Sweden). The detection limit for total (alpha+beta-type) tryptase in this assay was 1 ng/mL. The median serum tryptase level in healthy controls amounts to 5.6±2.8 ng/mL (range: 0-15 ng/mL) [48].

### Mast cell differentiation assay

Ficoll-isolated cord blood (CB) mononuclear cells (MNC, 1×10^6^/ml) were cultured in 24-well plates in RPMI 1640 medium containing 10% FCS, SCF (100 ng/ml) and IL-6 (100 ng/ml). Cultures were maintained with or without various concentrations of imatinib (0.001-1 μM). After 2 weeks, medium, cytokines and imatinib were replaced. After 4 weeks, cells were recovered and examined for the percentage of MC by Wright-Giemsa-staining, for their histamine- and tryptase content (after freeze-thawing), and for expression of tryptase- and KIT mRNA levels by qPCR. Histamine concentrations were determined by RIA and total tryptase concentrations by FEIA.

### Immunohistochemical staining of BM sections

The indirect immunoperoxidase staining technique was performed with serial sections (2 μM) of formalin-fixed and paraffin-embedded BM as described [49-51]. For MC detection and enumeration, antibodies against KIT (CD117) (polyclonal) and tryptase (mAb G3) were applied overnight. Slides were then washed and incubated with biotinylated anti-rabbit IgG or anti-mouse IgG, washed, and then exposed to streptavidin-biotin-peroxidase-complex. AEC was used as chromogen. The numbers of tryptase+ cells and KIT+ cells (MC) were determined using an Olympus BX50F4 microscope connected to a DP21 camera (Olympus, Hamburg, Germany) and expressed as percent of nucleated BM cells. We also confirmed the presence of MC by Giemsa staining and counted the numbers of MC on Giemsa-stained BM sections in our CML patients.

### Quantitative PCR (qPCR)

KIT- and tryptase mRNA levels were quantified in patient-derived BM MNC and cultured CB MNC-derived MC by qPCR essentially as described [52]. PCR primers used in this study are shown in Supplementary Table S1. Expression of KIT- and tryptase mRNA was quantified on a 7900HT Fast Real-Time PCR System (Applied Biosystems, Foster City, CA) using iTaq SYBR Green Supermix with ROX from Bio-Rad (Hercules, CA). KIT mRNA levels and tryptase mRNA levels were expressed as percent of ABL. In clinical follow-up samples, BCR/ABL1 mRNA levels were adjusted according to the international scale (IS) [53]. Conventional karyotyping and fluorescence in situ-hybridization (FISH) were performed according to published protocols [54].

### Treatment of C57BL/6J mice and BALB/c mice with imatinib

Two different mouse strains were examined, C57BL/6J mice obtained from the central animal facility of the University of Cologne (Cologne, Germany) and BALB/c mice purchased from IFFA-CREDO (Saint-Germain Sur L'arbresle, France). All animal experiments were approved by the local ethics committees for animal research of the University of Cologne and the Ecole Normale Supérieure de Cachan (Cachan, France). Both groups of mice were treated with imatinib by intraperitoneal (i.p.) injection. C57BL/6J mice (8-12 week-old) were treated with imatinib (60 mg/kg per day) or vehicle control (10 mice per group) for 42 days. After 21 days and 42 days, animals (10 each time point) were sacrificed. In each group of animals, the skin (back skin and ear skin), gastric submucosa, and splenic tissues were fixed in formalin and embedded in paraffin. Tissue sections were then examined for the presence and percentage of MC by Toludine Blue (TB) staining. The percentage of TB+ cells (MC) among all nucleated cells was determined by counting cells in five different high power fields (HPF). In a separate set of experiments, twenty-four 6 week-old BALB/c mice were divided into two groups of 12 mice each. One group received 25 mg/kg imatinib (dissolved in 0.5 mL PBS) twice daily by i.p. injection for 10 consecutive days, and the second (control) group of mice received vehicel control. On days 10, 17, 24, and 31, each 3 mice were sacrificed and peritoneal cells collected after i.p. injection of pre-warmed (37°C) phenol-red-free RPMI 1640 medium (Gibco Laboratories, Grand Island, NY). Peritoneal cell suspensions were examined for the percentage of MC by TB staining (on a hematocytometer) and for the levels of histamine. For determining histamine levels, 10^6^ peritoneal cells were centrifuged and resuspended in 1 ml of phenol red-free RPMI 1640 medium. Cell suspensions were then subjected to freeze-thawing, and histamine concentrations were determined by an automated flow-fluorometric technique as described [55]. Total histamine content in peritoneal cell suspensions was expressed in ng per 10^6^ cells.

### Statistical analysis

Significance levels were calculated by standard tests, including the Student´s t-test and analysis of variance (ANOVA). Differences were considered significant when p<0.05.

## SUPPLEMENTARY MATERIAL, FIGURES, TABLES


