# Supplementary material for: Long-term treatment with imatinib results in profound mast cell deficiency in Ph+ chronic myeloid leukemia
Source: Oncotarget. 2014 Dec 26;6(5):3071–84. doi: 10.18632/oncotarget.3074 (PMC4413638; doi:10.18632/oncotarget.3074)
Supplement: Supplementary file 1 [file oncotarget-06-3071-s001.pdf]

Long-term treatment with imatinib results in profound mast cell deficiency in Ph+ chronic myeloid leukemia

Supplemental Material

Tables

Supplemental Table S1

Oligonucleotide primer sequences for quantitative PCR

Gene	Primer Sequence
Tryptase -fwd	CGGGAACACCCGGAGGGACT
Tryptase -rev	GCCTGCAGCCAGGTGCCATT
CD117 -fwd	GCGTTCTGCTCCTACTGCTTCG
CD117 -rev	CCCTGGACTCACAGATGGTTGA
ABL -fwd	TGTATGATTTTGTGGCCAGTGGAG
ABL -rev	GCCTAAGACCCGGAGCTTTTCA
ABL/EXON1- fwd	GCTGTCCTCGTCCTCCAGCTGTT
ABL/EXON1- rev	TCAGATGCTACTGGCCGCTGAAG

CD, cluster of differentiation; fwd, forward; rev, reverse.

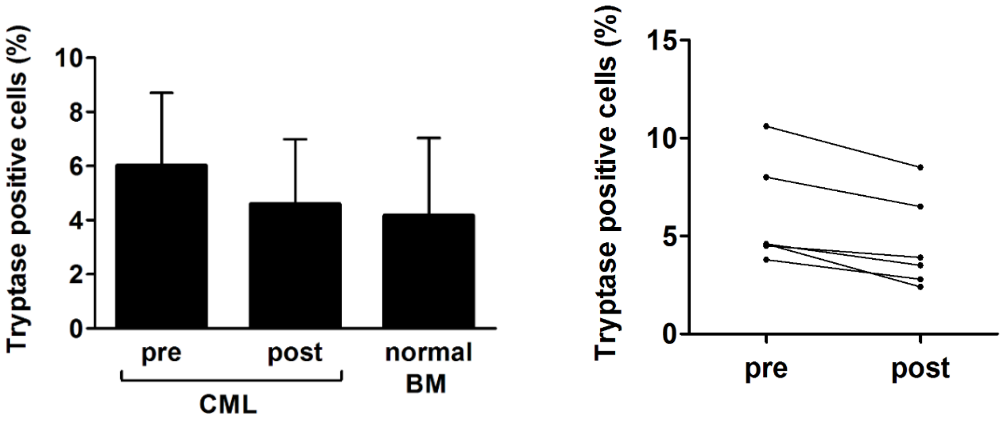

Cerny-Reiterer et al, Supplemental Figure 1A

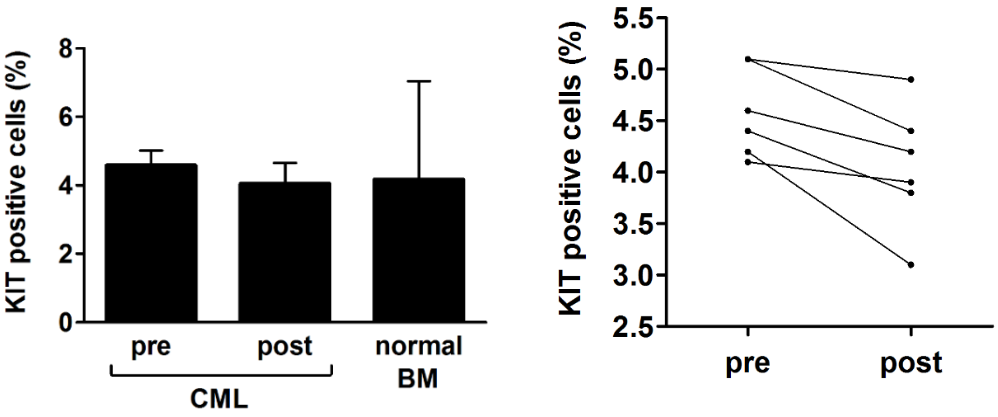

Cerny-Reiterer et al, Supplemental Figure 1B

Supplemental Figure S1: Effects of imatinib on mast cell (MC) numbers in the bone marrow of patients with CML within the first 12 months. Bone marrow (BM) biopsy material was obtained from BM sections of CML patients (n=6) at diagnosis (pre) and after (post) treatment with imatinib (400 mg/day) for less than 1 year. In addition, control BM section from 5 patients were examined. Serial sections were prepared from paraffin-embedded BM specimens and stained with antibodies against tryptase (A) or KIT (B) by indirect immunohistochemistry. The percentages of tryptase+ MC and KIT+ MC relative to all nucleated BM cells (500 cells counted) was determined using an Olympus AX-1 microscope equipped with a 100x/1.35 UPlan-Apo objective lens. Results in the left panels represent the mean±S.D. (percent-values) from all donors before and after therapy, and a comparison to normal control BM samples (n=5). The right panels show the percentages of tryptase+ MC (A) or KIT+ MC (B) in each individual patient before (pre) and after (post) therapy.
